# Ethyl 2-(4-bromo­phen­yl)-1-*sec*-butyl-1*H*-benzimidazole-5-carboxyl­ate

**DOI:** 10.1107/S1600536811048999

**Published:** 2011-11-30

**Authors:** Natarajan Arumugam, Nurziana Ngah, Shafida Abd Hamid, Aisyah Saad Abdul Rahim

**Affiliations:** a School of Pharmaceutical Sciences, Universiti Sains Malaysia, 11800 USM, Penang, Malaysia; b Kulliyyah of Science, International Islamic University Malaysia, Bandar Indera Mahkota, 25200 Kuantan, Pahang, Malaysia

## Abstract

In the title compound, C_20_H_21_BrN_2_O_2_, the bromo­phenyl ring is twisted by 40.13 (8)° from the benzimidazole mean plane and the Br atom deviates by 0.753 (1) Å from that plane. The *sec*-butyl group is disordered over two conformations in a 0.898 (5):0.102 (5) ratio. In the crystal, mol­ecules related by translation along [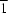
10] are linked into chains *via* weak C—H⋯Br hydrogen bonds.

## Related literature

For the synthesis and closely related structures, see: Arumugam *et al.* (2010 ▶, 2011 ▶); Navarrete-Vazquez *et al.* (2006 ▶). For therapeutic properties of benzimidazole derivatives, see: Vitale *et al.* (2008 ▶, 2009 ▶); Arienti *et al.* (2005 ▶). For standard bond lengths, see: Allen *et al.* (1987 ▶). For the low-temperature device used in the data collection, see: Cosier & Glazer (1986 ▶).
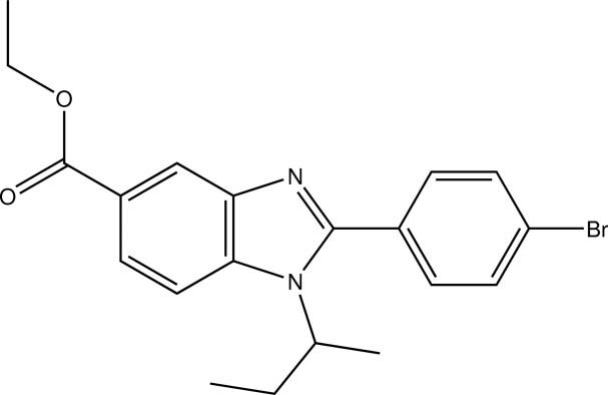


## Experimental

### 

#### Crystal data

C_20_H_21_BrN_2_O_2_
*M* *_r_* = 401.30
Monoclinic, 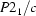

*a* = 10.5187 (2) Å
*b* = 12.7525 (2) Å
*c* = 13.7444 (2) Å
β = 98.101 (1)°
*V* = 1825.27 (5) Å^3^
*Z* = 4
Mo *K*α radiation
μ = 2.27 mm^−1^
*T* = 100 K
0.39 × 0.39 × 0.20 mm

#### Data collection

Bruker SMART APEXII CCD area-detector diffractometer
Absorption correction: multi-scan (*SADABS*; Bruker, 2009 ▶) *T*_min_ = 0.471, *T*_max_ = 0.666
24453 measured reflections
3221 independent reflections
3073 reflections with *I* > 2σ(*I*)
*R*_int_ = 0.024

#### Refinement

*R*[*F* ^2^ > 2σ(*F* ^2^)] = 0.022
*wR*(*F* ^2^) = 0.056
*S* = 1.08
3221 reflections
248 parameters
12 restraints
H-atom parameters constrained
Δρ_max_ = 0.30 e Å^−3^
Δρ_min_ = −0.24 e Å^−3^

### 

Data collection: *APEX2* (Bruker, 2009 ▶); cell refinement: *SAINT* (Bruker, 2009 ▶); data reduction: *SAINT*; program(s) used to solve structure: *SHELXTL* (Sheldrick, 2008 ▶); program(s) used to refine structure: *SHELXTL*; molecular graphics: *SHELXTL*; software used to prepare material for publication: *SHELXTL* and *PLATON* (Spek, 2009 ▶).

## Supplementary Material

Crystal structure: contains datablock(s) global, I. DOI: 10.1107/S1600536811048999/cv5201sup1.cif

Structure factors: contains datablock(s) I. DOI: 10.1107/S1600536811048999/cv5201Isup2.hkl

Supplementary material file. DOI: 10.1107/S1600536811048999/cv5201Isup3.cml

Additional supplementary materials:  crystallographic information; 3D view; checkCIF report

## Figures and Tables

**Table 1:** Hydrogen-bond geometry (Å, °)

*D*—H⋯*A*	*D*—H	H⋯*A*	*D*⋯*A*	*D*—H⋯*A*
C16—H16*A*⋯Br1^i^	0.98	2.79	3.533 (2)	133

Symmetry code: (i) 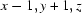.

**Fig. 1.**
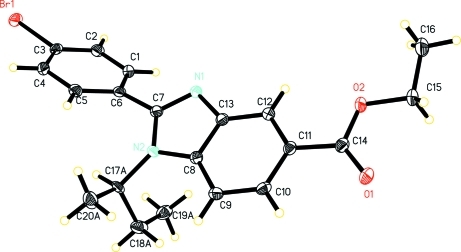
The molecular structure of (I) with displacement ellipsods drawn at the 50% probability level. The minor component of disordered fragment has been omitted.

**Fig. 2.**
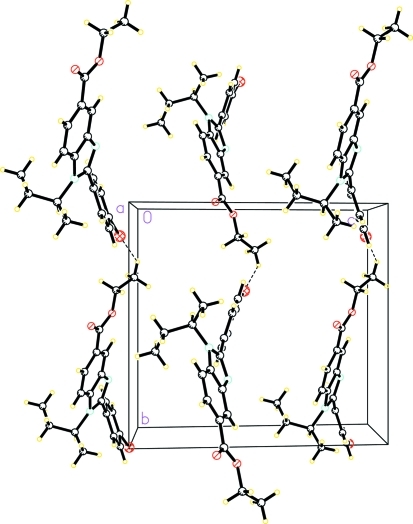
The molecular packing of (I) viewed down the a axis. The minor component of disorder has been omitted for clarity.

## supplementary crystallographic information

### Comment

Accelerated condensation of substituted phenylenediamines with adducts of aldehydes under microwave conditions provides access into 2-arylbenzimidazoles (Navarrete-Vazquez *et al.*, 2006; Arumugam *et al.*, 2010; 2011). These 2-substituted benzimidazoles have recently gained attention due to their antiviral and antiproliferative activities (Vitale *et al.*, 2008; 2009). Not only that, a series of novel 2-arylbenzimidazoles was found to exhibit highly selective inhibition on chk2 kinase, which helps to control DNA damage and could prove useful as an adjuvant to radiotherapy (Arienti *et al.*, 2005). In continuation with our work in 2-arylbenzimidazoles (Arumugam *et al.*, 2010; 2011), we present herein the X-ray crystal structure determination of the title compound.

The title compound, (Fig. 1), is similar to those previously reported, ethyl 1-*sec*-butyl-2-(4-chlorophenyl)-1*H*-benzimidazole-5- carboxylate (Arumugam *et al.*, 2010) and ethyl 1-*sec*-butyl-2- (4-fluorophenyl)-1*H*-benzimidazole-5-carboxylate (Arumugam *et al.*, 2011), except the bromine atom is attached at the *para* position of benzene ring.The bond lengths and angles are in normal ranges (Allen *et al.*, 1987) and in agreement with those reported by Arumugam *et al.* (2010) and Arumugam *et al.* (2011). The *sec*-butyl group (C17/C18/C19/C20) is disordered over two conformations in a ratio 0.898 (5):0.102 (5). The bromophenyl ring (C1—C6/Br1) is twisted at 40.13 (8)° from the benzimidazole mean plane (C8/C9/C10/C11/C12/C13/N1/N2/C7) and Br atom deviates at 0.753 (1)Å from that plane.

In the crystal structure (Fig. 2), the molecules related by translation along [-110] are linked into chains *via* weak intermolecular C16—H16A···Br1 hydrogen bonds (Table 1).

### Experimental

Preparation of the title compound was performed using the previous procedure described by Arumugam *et al.* (2010) and Arumugam *et al.* (2011). Recrystallization of the crude product from ethyl acetate furnished colourless crystals suitable for X-ray analysis.

### Refinement

X-ray data were collected at low temperature (Cosier & Glazer, 1986). All H atoms were positioned geometrically and refined using riding model with C—H = 0.95–1.00Å and *U*_iso_(H)=1.2 or 1.5*U*_eq_(C). A *sec*- butyl group (C17/C18/C19/C20) is disordered over two conformations in a ratio 0.898 (5):0.102 (5). A minor component of disorder (C17B/C18B/C19B/C20B) was refined isotropically. A rotating group model was applied for methyl group.

### Crystal data

**Table:**

C_20_H_21_BrN_2_O_2_	*F*(000) = 824
*M**_r_* = 401.30	*D*_x_ = 1.460 Mg m^−^^3^
Monoclinic, *P*2_1_/*c*	Mo *K*α radiation, λ = 0.71073 Å
Hall symbol: -P 2ybc	Cell parameters from 15956 reflections
*a* = 10.5187 (2) Å	θ = 1.9–25.0°
*b* = 12.7525 (2) Å	µ = 2.27 mm^−^^1^
*c* = 13.7444 (2) Å	*T* = 100 K
β = 98.101 (1)°	Block, colourless
*V* = 1825.27 (5) Å^3^	0.39 × 0.39 × 0.20 mm
*Z* = 4	

### Data collection

**Table:**

Bruker SMART APEXII CCD area-detector diffractometer	3221 independent reflections
Radiation source: fine-focus sealed tube	3073 reflections with *I* > 2σ(*I*)
graphite	*R*_int_ = 0.024
Detector resolution: 83.66 pixels mm^-1^	θ_max_ = 25.0°, θ_min_ = 1.9°
φ and ω scans	*h* = −12→12
Absorption correction: multi-scan (*SADABS*; Bruker, 2009)	*k* = −15→15
*T*_min_ = 0.471, *T*_max_ = 0.666	*l* = −16→16
24453 measured reflections	

### Refinement

**Table:**

Refinement on *F*^2^	Primary atom site location: structure-invariant direct methods
Least-squares matrix: full	Secondary atom site location: difference Fourier map
*R*[*F*^2^ > 2σ(*F*^2^)] = 0.022	Hydrogen site location: inferred from neighbouring sites
*wR*(*F*^2^) = 0.056	H-atom parameters constrained
*S* = 1.08	*w* = 1/[σ^2^(*F*_o_^2^) + (0.0253*P*)^2^ + 1.2079*P*] where *P* = (*F*_o_^2^ + 2*F*_c_^2^)/3
3221 reflections	(Δ/σ)_max_ < 0.001
248 parameters	Δρ_max_ = 0.30 e Å^−^^3^
12 restraints	Δρ_min_ = −0.24 e Å^−^^3^

### Special details

**Table:**

Experimental. The crystal was placed in the cold stream of an Oxford Cryosystems Cobra open=flow nitrogen cryostat (Cosier & Glazer, 1986) operating at 100.0 (1) K.
Geometry. All e.s.d.'s (except the e.s.d. in the dihedral angle between two l.s. planes) are estimated using the full covariance matrix. The cell e.s.d.'s are taken into account individually in the estimation of e.s.d.'s in distances, angles and torsion angles; correlations between e.s.d.'s in cell parameters are only used when they are defined by crystal symmetry. An approximate (isotropic) treatment of cell e.s.d.'s is used for estimating e.s.d.'s involving l.s. planes.
Refinement. Refinement of *F*^2^ against ALL reflections. The weighted *R*-factor *wR* and goodness of fit *S* are based on *F*^2^, conventional *R*-factors *R* are based on *F*, with *F* set to zero for negative *F*^2^. The threshold expression of *F*^2^ > σ(*F*^2^) is used only for calculating *R*-factors(gt) *etc*. and is not relevant to the choice of reflections for refinement. *R*-factors based on *F*^2^ are statistically about twice as large as those based on *F*, and *R*- factors based on ALL data will be even larger.

### Fractional atomic coordinates and isotropic or equivalent isotropic displacement parameters (Å^2^)

**Table:**

	*x*	*y*	*z*	*U*_iso_*/*U*_eq_	Occ. (<1)
Br1	1.437653 (17)	−0.132336 (14)	0.052952 (13)	0.02810 (7)	
O1	0.49686 (12)	0.48183 (10)	0.16898 (9)	0.0271 (3)	
O2	0.66303 (12)	0.53232 (9)	0.09400 (9)	0.0257 (3)	
N1	0.97007 (13)	0.22220 (11)	0.12388 (10)	0.0189 (3)	
N2	0.87714 (13)	0.08790 (11)	0.19561 (10)	0.0196 (3)	
C1	1.21317 (17)	0.10333 (14)	0.14588 (12)	0.0216 (4)	
H1A	1.2249	0.1737	0.1680	0.026*	
C2	1.31726 (17)	0.04696 (14)	0.12213 (12)	0.0224 (4)	
H2A	1.4001	0.0780	0.1278	0.027*	
C3	1.29818 (16)	−0.05549 (14)	0.08999 (12)	0.0212 (4)	
C4	1.17921 (17)	−0.10309 (14)	0.08218 (12)	0.0222 (4)	
H4A	1.1684	−0.1738	0.0608	0.027*	
C5	1.07572 (17)	−0.04590 (13)	0.10604 (12)	0.0211 (4)	
H5A	0.9933	−0.0777	0.1008	0.025*	
C6	1.09155 (16)	0.05814 (13)	0.13774 (11)	0.0190 (3)	
C7	0.98140 (16)	0.12428 (13)	0.15413 (12)	0.0187 (3)	
C8	0.79159 (16)	0.17113 (13)	0.19091 (12)	0.0191 (3)	
C9	0.67050 (16)	0.18299 (14)	0.22094 (12)	0.0212 (4)	
H9A	0.6311	0.1276	0.2520	0.025*	
C10	0.61127 (16)	0.27893 (14)	0.20317 (12)	0.0204 (3)	
H10A	0.5289	0.2896	0.2223	0.024*	
C11	0.66927 (16)	0.36167 (13)	0.15738 (12)	0.0189 (3)	
C12	0.78936 (16)	0.34958 (13)	0.12819 (12)	0.0185 (3)	
H12A	0.8283	0.4051	0.0970	0.022*	
C13	0.85105 (16)	0.25334 (13)	0.14619 (12)	0.0178 (3)	
C14	0.59928 (16)	0.46288 (13)	0.14229 (12)	0.0204 (4)	
C15	0.60340 (19)	0.63412 (14)	0.07495 (14)	0.0282 (4)	
H15A	0.6069	0.6746	0.1367	0.034*	
H15B	0.5124	0.6260	0.0457	0.034*	
C16	0.6779 (2)	0.68923 (16)	0.00444 (17)	0.0381 (5)	
H16A	0.6410	0.7589	−0.0107	0.057*	
H16B	0.6736	0.6482	−0.0563	0.057*	
H16C	0.7677	0.6965	0.0343	0.057*	
C17A	0.8740 (2)	−0.00625 (16)	0.25876 (16)	0.0213 (5)	0.898 (5)
H17A	0.9573	−0.0439	0.2583	0.026*	0.898 (5)
C18A	0.8681 (3)	0.0262 (2)	0.36369 (17)	0.0287 (6)	0.898 (5)
H18A	0.8781	−0.0366	0.4065	0.034*	0.898 (5)
H18B	0.7829	0.0574	0.3683	0.034*	0.898 (5)
C19A	0.9724 (2)	0.10532 (19)	0.39981 (15)	0.0306 (6)	0.898 (5)
H19A	0.9687	0.1218	0.4690	0.046*	0.898 (5)
H19B	0.9591	0.1695	0.3605	0.046*	0.898 (5)
H19C	1.0567	0.0756	0.3932	0.046*	0.898 (5)
C20A	0.7673 (2)	−0.08225 (17)	0.21800 (19)	0.0308 (6)	0.898 (5)
H20A	0.7736	−0.0974	0.1489	0.046*	0.898 (5)
H20B	0.6836	−0.0505	0.2230	0.046*	0.898 (5)
H20C	0.7762	−0.1475	0.2559	0.046*	0.898 (5)
C17B	0.9167 (17)	0.0113 (12)	0.2755 (10)	0.065 (13)*	0.102 (5)
H17B	0.9988	−0.0258	0.2688	0.077*	0.102 (5)
C18B	0.9091 (19)	0.0503 (16)	0.3795 (13)	0.017 (5)*	0.102 (5)
H18C	0.9461	−0.0025	0.4271	0.025*	0.102 (5)
H18D	0.8191	0.0623	0.3873	0.025*	0.102 (5)
H18E	0.9572	0.1160	0.3909	0.025*	0.102 (5)
C19B	0.8038 (18)	−0.0613 (15)	0.2757 (13)	0.032 (5)*	0.102 (5)
H19D	0.7256	−0.0199	0.2814	0.038*	0.102 (5)
H19E	0.8194	−0.1090	0.3329	0.038*	0.102 (5)
C20B	0.784 (2)	−0.1251 (15)	0.1810 (12)	0.029 (5)*	0.102 (5)
H20D	0.7084	−0.1703	0.1806	0.043*	0.102 (5)
H20E	0.8599	−0.1685	0.1771	0.043*	0.102 (5)
H20F	0.7704	−0.0776	0.1245	0.043*	0.102 (5)

### Atomic displacement parameters (Å^2^)

**Table:**

	*U*^11^	*U*^22^	*U*^33^	*U*^12^	*U*^13^	*U*^23^
Br1	0.02917 (11)	0.02848 (11)	0.02913 (11)	0.01432 (7)	0.01271 (8)	0.00389 (7)
O1	0.0221 (6)	0.0268 (7)	0.0341 (7)	0.0081 (5)	0.0097 (5)	0.0009 (5)
O2	0.0261 (7)	0.0199 (6)	0.0332 (7)	0.0098 (5)	0.0111 (5)	0.0060 (5)
N1	0.0185 (7)	0.0185 (7)	0.0205 (7)	0.0036 (6)	0.0059 (6)	0.0023 (6)
N2	0.0205 (7)	0.0177 (7)	0.0221 (7)	0.0038 (6)	0.0079 (6)	0.0056 (6)
C1	0.0252 (9)	0.0180 (8)	0.0225 (8)	0.0037 (7)	0.0061 (7)	0.0018 (7)
C2	0.0200 (8)	0.0245 (9)	0.0235 (9)	0.0033 (7)	0.0056 (7)	0.0042 (7)
C3	0.0230 (9)	0.0240 (9)	0.0178 (8)	0.0111 (7)	0.0073 (7)	0.0044 (7)
C4	0.0292 (9)	0.0183 (8)	0.0193 (8)	0.0050 (7)	0.0044 (7)	0.0015 (7)
C5	0.0219 (9)	0.0207 (8)	0.0208 (8)	0.0020 (7)	0.0038 (7)	0.0034 (7)
C6	0.0210 (8)	0.0207 (8)	0.0163 (8)	0.0050 (7)	0.0060 (6)	0.0054 (6)
C7	0.0190 (8)	0.0199 (9)	0.0177 (8)	0.0024 (7)	0.0045 (6)	0.0017 (6)
C8	0.0202 (8)	0.0193 (8)	0.0181 (8)	0.0032 (7)	0.0039 (6)	0.0017 (6)
C9	0.0211 (9)	0.0226 (9)	0.0212 (8)	0.0008 (7)	0.0072 (7)	0.0030 (7)
C10	0.0172 (8)	0.0264 (9)	0.0184 (8)	0.0031 (7)	0.0054 (6)	−0.0002 (7)
C11	0.0204 (8)	0.0198 (8)	0.0162 (8)	0.0033 (7)	0.0017 (6)	−0.0015 (6)
C12	0.0200 (8)	0.0178 (8)	0.0182 (8)	0.0013 (6)	0.0041 (6)	0.0008 (6)
C13	0.0178 (8)	0.0196 (8)	0.0164 (8)	0.0021 (7)	0.0033 (6)	0.0002 (6)
C14	0.0207 (9)	0.0225 (9)	0.0178 (8)	0.0037 (7)	0.0018 (7)	−0.0018 (7)
C15	0.0311 (10)	0.0209 (9)	0.0341 (10)	0.0121 (7)	0.0099 (8)	0.0054 (7)
C16	0.0394 (12)	0.0276 (11)	0.0508 (13)	0.0134 (9)	0.0183 (10)	0.0105 (9)
C17A	0.0196 (12)	0.0163 (10)	0.0300 (11)	0.0046 (9)	0.0106 (9)	0.0119 (8)
C18A	0.0251 (13)	0.0367 (14)	0.0253 (11)	0.0060 (11)	0.0067 (10)	0.0112 (10)
C19A	0.0321 (12)	0.0402 (13)	0.0197 (10)	0.0122 (10)	0.0039 (8)	0.0018 (9)
C20A	0.0260 (11)	0.0210 (11)	0.0469 (15)	−0.0032 (9)	0.0106 (10)	0.0020 (11)

### Geometric parameters (Å, °)

**Table:**

Br1—C3	1.8927 (16)	C15—C16	1.504 (3)
O1—C14	1.210 (2)	C15—H15A	0.9900
O2—C14	1.342 (2)	C15—H15B	0.9900
O2—C15	1.450 (2)	C16—H16A	0.9800
N1—C7	1.316 (2)	C16—H16B	0.9800
N1—C13	1.388 (2)	C16—H16C	0.9800
N2—C7	1.385 (2)	C17A—C18A	1.510 (3)
N2—C8	1.387 (2)	C17A—C20A	1.528 (4)
N2—C17B	1.484 (5)	C17A—H17A	1.0000
N2—C17A	1.485 (2)	C18A—C19A	1.522 (4)
C1—C2	1.387 (2)	C18A—H18A	0.9900
C1—C6	1.393 (2)	C18A—H18B	0.9900
C1—H1A	0.9500	C19A—H19A	0.9800
C2—C3	1.385 (3)	C19A—H19B	0.9800
C2—H2A	0.9500	C19A—H19C	0.9800
C3—C4	1.381 (3)	C20A—H20A	0.9800
C4—C5	1.387 (2)	C20A—H20B	0.9800
C4—H4A	0.9500	C20A—H20C	0.9800
C5—C6	1.399 (2)	C17B—C19B	1.507 (6)
C5—H5A	0.9500	C17B—C18B	1.526 (6)
C6—C7	1.476 (2)	C17B—H17B	1.0000
C8—C9	1.401 (2)	C18B—H18C	0.9800
C8—C13	1.406 (2)	C18B—H18D	0.9800
C9—C10	1.379 (2)	C18B—H18E	0.9800
C9—H9A	0.9500	C19B—C20B	1.524 (6)
C10—C11	1.411 (2)	C19B—H19D	0.9900
C10—H10A	0.9500	C19B—H19E	0.9900
C11—C12	1.387 (2)	C20B—H20D	0.9800
C11—C14	1.486 (2)	C20B—H20E	0.9800
C12—C13	1.394 (2)	C20B—H20F	0.9800
C12—H12A	0.9500		
			
C14—O2—C15	116.53 (13)	O2—C14—C11	111.71 (14)
C7—N1—C13	104.23 (14)	O2—C15—C16	106.46 (14)
C7—N2—C8	105.69 (13)	O2—C15—H15A	110.4
C7—N2—C17B	111.6 (7)	C16—C15—H15A	110.4
C8—N2—C17B	130.6 (7)	O2—C15—H15B	110.4
C7—N2—C17A	126.56 (15)	C16—C15—H15B	110.4
C8—N2—C17A	125.33 (14)	H15A—C15—H15B	108.6
C17B—N2—C17A	20.4 (7)	C15—C16—H16A	109.5
C2—C1—C6	120.87 (16)	C15—C16—H16B	109.5
C2—C1—H1A	119.6	H16A—C16—H16B	109.5
C6—C1—H1A	119.6	C15—C16—H16C	109.5
C3—C2—C1	118.72 (16)	H16A—C16—H16C	109.5
C3—C2—H2A	120.6	H16B—C16—H16C	109.5
C1—C2—H2A	120.6	N2—C17A—C18A	110.12 (18)
C4—C3—C2	121.90 (16)	N2—C17A—C20A	111.97 (19)
C4—C3—Br1	118.66 (13)	C18A—C17A—C20A	113.38 (19)
C2—C3—Br1	119.43 (13)	N2—C17A—H17A	107.0
C3—C4—C5	118.88 (16)	C18A—C17A—H17A	107.0
C3—C4—H4A	120.6	C20A—C17A—H17A	107.0
C5—C4—H4A	120.6	C17A—C18A—C19A	111.61 (19)
C4—C5—C6	120.61 (16)	C17A—C18A—H18A	109.3
C4—C5—H5A	119.7	C19A—C18A—H18A	109.3
C6—C5—H5A	119.7	C17A—C18A—H18B	109.3
C1—C6—C5	119.01 (15)	C19A—C18A—H18B	109.3
C1—C6—C7	118.89 (15)	H18A—C18A—H18B	108.0
C5—C6—C7	121.87 (15)	N2—C17B—C19B	105.4 (13)
N1—C7—N2	113.96 (14)	N2—C17B—C18B	115.6 (14)
N1—C7—C6	122.14 (15)	C19B—C17B—C18B	93.0 (3)
N2—C7—C6	123.74 (14)	N2—C17B—H17B	113.6
N2—C8—C9	132.80 (16)	C19B—C17B—H17B	113.6
N2—C8—C13	105.39 (14)	C18B—C17B—H17B	113.6
C9—C8—C13	121.81 (15)	C17B—C18B—H18C	109.5
C10—C9—C8	116.76 (15)	C17B—C18B—H18D	109.5
C10—C9—H9A	121.6	H18C—C18B—H18D	109.5
C8—C9—H9A	121.6	C17B—C18B—H18E	109.5
C9—C10—C11	121.99 (15)	H18C—C18B—H18E	109.5
C9—C10—H10A	119.0	H18D—C18B—H18E	109.5
C11—C10—H10A	119.0	C17B—C19B—C20B	109.9 (15)
C12—C11—C10	120.98 (15)	C17B—C19B—H19D	109.7
C12—C11—C14	120.65 (15)	C20B—C19B—H19D	109.7
C10—C11—C14	118.37 (15)	C17B—C19B—H19E	109.7
C11—C12—C13	117.83 (15)	C20B—C19B—H19E	109.7
C11—C12—H12A	121.1	H19D—C19B—H19E	108.2
C13—C12—H12A	121.1	C19B—C20B—H20D	109.5
N1—C13—C12	128.63 (15)	C19B—C20B—H20E	109.5
N1—C13—C8	110.73 (14)	H20D—C20B—H20E	109.5
C12—C13—C8	120.63 (15)	C19B—C20B—H20F	109.5
O1—C14—O2	123.13 (15)	H20D—C20B—H20F	109.5
O1—C14—C11	125.15 (16)	H20E—C20B—H20F	109.5
			
C6—C1—C2—C3	0.0 (3)	C10—C11—C12—C13	−0.4 (2)
C1—C2—C3—C4	−0.8 (3)	C14—C11—C12—C13	178.76 (15)
C1—C2—C3—Br1	177.88 (12)	C7—N1—C13—C12	−178.63 (17)
C2—C3—C4—C5	0.9 (3)	C7—N1—C13—C8	0.12 (18)
Br1—C3—C4—C5	−177.81 (12)	C11—C12—C13—N1	179.76 (16)
C3—C4—C5—C6	−0.2 (2)	C11—C12—C13—C8	1.1 (2)
C2—C1—C6—C5	0.6 (2)	N2—C8—C13—N1	−0.08 (18)
C2—C1—C6—C7	−173.87 (15)	C9—C8—C13—N1	179.70 (15)
C4—C5—C6—C1	−0.6 (2)	N2—C8—C13—C12	178.78 (15)
C4—C5—C6—C7	173.78 (15)	C9—C8—C13—C12	−1.4 (3)
C13—N1—C7—N2	−0.12 (19)	C15—O2—C14—O1	0.4 (2)
C13—N1—C7—C6	175.47 (15)	C15—O2—C14—C11	179.89 (14)
C8—N2—C7—N1	0.07 (19)	C12—C11—C14—O1	−176.80 (16)
C17B—N2—C7—N1	−146.9 (8)	C10—C11—C14—O1	2.4 (3)
C17A—N2—C7—N1	−162.89 (18)	C12—C11—C14—O2	3.7 (2)
C8—N2—C7—C6	−175.44 (15)	C10—C11—C14—O2	−177.09 (14)
C17B—N2—C7—C6	37.6 (8)	C14—O2—C15—C16	−169.26 (16)
C17A—N2—C7—C6	21.6 (3)	C7—N2—C17A—C18A	108.4 (2)
C1—C6—C7—N1	38.2 (2)	C8—N2—C17A—C18A	−51.4 (3)
C5—C6—C7—N1	−136.19 (17)	C17B—N2—C17A—C18A	61 (2)
C1—C6—C7—N2	−146.68 (16)	C7—N2—C17A—C20A	−124.5 (2)
C5—C6—C7—N2	39.0 (2)	C8—N2—C17A—C20A	75.7 (2)
C7—N2—C8—C9	−179.74 (18)	C17B—N2—C17A—C20A	−172 (2)
C17B—N2—C8—C9	−41.6 (10)	N2—C17A—C18A—C19A	−51.2 (2)
C17A—N2—C8—C9	−16.5 (3)	C20A—C17A—C18A—C19A	−177.58 (17)
C7—N2—C8—C13	0.01 (17)	C7—N2—C17B—C19B	−150.3 (8)
C17B—N2—C8—C13	138.2 (10)	C8—N2—C17B—C19B	73.4 (14)
C17A—N2—C8—C13	163.24 (19)	C17A—N2—C17B—C19B	−9.8 (15)
N2—C8—C9—C10	−179.33 (17)	C7—N2—C17B—C18B	108.5 (9)
C13—C8—C9—C10	1.0 (2)	C8—N2—C17B—C18B	−27.8 (16)
C8—C9—C10—C11	−0.2 (2)	C17A—N2—C17B—C18B	−111 (2)
C9—C10—C11—C12	0.0 (3)	N2—C17B—C19B—C20B	66.5 (18)
C9—C10—C11—C14	−179.21 (15)	C18B—C17B—C19B—C20B	−175.9 (16)

### Hydrogen-bond geometry (Å, °)

**Table:**

*D*—H···*A*	*D*—H	H···*A*	*D*···*A*	*D*—H···*A*
C16—H16A···Br1^i^	0.98	2.79	3.533 (2)	133

Symmetry codes: (i) *x*−1, *y*+1, *z*.
